# Focal Adhesion Kinase Plays a Role in Osteoblast Mechanotransduction *In Vitro* but Does Not Affect Load-Induced Bone Formation *In Vivo*

**DOI:** 10.1371/journal.pone.0043291

**Published:** 2012-09-21

**Authors:** Alesha B. Castillo, Jennifer T. Blundo, Julia C. Chen, Kristen L. Lee, Nikitha Reddy Yereddi, Eugene Jang, Shefali Kumar, W. Joyce Tang, Sarah Zarrin, Jae-Beom Kim, Christopher R. Jacobs

**Affiliations:** 1 Department of Rehabilitation Research and Development, Center for Tissue Regeneration, Repair, and Restoration, Veterans Affairs Palo Alto Health Care System, Palo Alto, California, United States of America; 2 Department of Surgery, Stanford University School of Medicine, Stanford, California, United States of America; 3 Department of Mechanical Engineering, Stanford University, Stanford, California, United States of America; 4 Department of Biomedical Engineering, Columbia University, New York, New York, United States of America; Universidade de São Paulo, Brazil

## Abstract

A healthy skeleton relies on bone's ability to respond to external mechanical forces. The molecular mechanisms by which bone cells sense and convert mechanical stimuli into biochemical signals, a process known as mechanotransduction, are unclear. Focal adhesions play a critical role in cell survival, migration and sensing physical force. Focal adhesion kinase (FAK) is a non-receptor protein tyrosine kinase that controls focal adhesion dynamics and can mediate reparative bone formation *in vivo* and osteoblast mechanotransduction *in vitro*. Based on these data, we hypothesized that FAK plays a role in load-induced bone formation. To test this hypothesis, we performed *in vitro* fluid flow experiments and *in vivo* bone loading studies in FAK−/− clonal lines and conditional FAK knockout mice, respectively. FAK−/− osteoblasts showed an ablated prostaglandin E_2_ (PGE_2_) response to fluid flow shear. This effect was reversed with the re-expression of wild-type FAK. Re-expression of FAK containing site-specific mutations at Tyr-397 and Tyr-925 phosphorylation sites did not rescue the phenotype, suggesting that these sites are important in osteoblast mechanotransduction. Interestingly, mice in which FAK was conditionally deleted in osteoblasts and osteocytes did not exhibit altered load-induced periosteal bone formation. Together these data suggest that although FAK is important in mechanically-induced signaling in osteoblasts *in vitro*, it is not required for an adaptive response *in vivo*, possibly due to a compensatory mechanism that does not exist in the cell culture system.

## Introduction

Mechanical integrity of a healthy skeleton is maintained through the cell-based processes of modeling and remodeling, which are greatly influenced by external physical stimuli. The molecular mechanisms by which skeletal progenitors and bone cells sense and respond to mechanical cues is unclear, but it likely involves activation of force sensitive molecules or “mechanosensors” that, in turn, initiate an intracellular signaling cascade altering cell behavior and function. Focal adhesions are large dynamic complexes that link the extracellular matrix (ECM) through integrin-ECM binding to the intracellular cytoskeleton [1]. Transmembrane integrin heterodimers comprise the core transmembrane component of focal adhesions and bind directly to collagen and fibronectin, among other ECM molecules, via their extracellular domain. Their cytoplasmic tails bind cytoskeletal proteins including talin and paxillin, both of which link directly to the actin cytoskeleton. Indeed, integrins have been shown to be important in load-induced bone formation [2]; however, as integrins do not possess intrinsic enzymatic activity, focal adhesion-mediated signal transduction is carried out by associated molecules that initiate downstream signaling events including tyrosine phosphorylation [3], intracellular calcium release [4] and MAPK activation [5].

Focal adhesion kinase (FAK) is a 125 kD non-receptor tyrosine kinase that regulates focal adhesion dynamics [6], [7], [8], is required for anchorage-dependent cell survival [9], [10], and is important in cell migration, proliferation and survival [11]. The protein structure of FAK includes 3 major domains: the central kinase domain, the N-terminal FERM (protein 4.1, ezrin, radixin and moesin homology) domain, and the C-terminal FRNK (FAK-related-non-kinase) domain, which contains the focal adhesion targeting (FAT) domain. The N-terminal domain contains a major FAK autophosphorylation site at tyrosine 397 (Tyr-397), which has been shown to regulate cell motility [12], [13]. The C-terminal domain of focal adhesion kinase contains two tyrosine phosphorylation sites at Tyr-861 and Tyr-925 and four serine phosphorylation sites at Ser 722, Ser 840, Ser 843 and Ser 910 [14].

FAK been implicated in cellular mechanotransduction [15], [16], [17], including in bone cells [18]. Both bone and endothelial cells exposed to fluid flow shear stress *in vitro* exhibit increased FAK phosphorylation [18] and downstream MAPK activation [19], as compared to cells maintained in static culture. FAK phosphorylation has also been linked to NFκB activation [20] as well as calcium release via large conductance calcium channels [21]. Disruption of the FAK gene in osteoblasts *in vitro* leads to reductions in fluid flow-induced ERK phosphorylation, c-fos and Cox-2 expression, as well as prostaglandin E_2_ (PGE_2_) release [18], all of which are important signaling events for normal osteoblast function and bone formation [22], [23]. Ablation of the FAK gene in osteoblasts and osteocytes *in vivo* slows bone regeneration and interrupts the response of bone marrow cells to anabolic mechanical stimuli in a tibial injury model [24], [25]. Together, these data suggest that FAK plays an important role in osteoblast function; however, its role in mechanical adaptation of the skeleton *in vivo* is unclear. Furthermore, the precise phosphorylation sites on FAK essential to load-induced activation in bone cells have not been identified.

FAK relies on the focal adhesion associated proteins paxillin and talin to indirectly associate with integrins via their C-terminal domain binding sites [26]. Paxillin is an adaptor protein that binds vinculin and is phosphorylated by a range of growth factors, as well as by integrin activation [27]. Talin is a cytoskeletal protein that binds β integrin and activates focal adhesions [28]. Vinculin is a cytoskeletal protein that is part of the integrin-cytoskeletal protein assembly found at focal adhesions. It binds to several proteins including α-actinin, talin and paxillin, and is involved in integrin signaling [29]. Another protein that is an important mediator in integrin signaling events is proline-rich tyrosine kinase 2 (Pyk2). Pyk2 is a 116 kD cytoplasmic protein that has 45% sequence homology with FAK [30]. Overexpression of Pyk2 has been shown to result in apoptosis of fibroblast and epithelial cell lines [31], suggesting that it has an important role in mediating cell survival. Previous western blot analysis has shown increased expression of the activated versus the inactive form of Pyk2 in FAK−/− osteoblasts [32], suggesting co-dependence.

Based on previous studies, we hypothesized that FAK mediates load-induced bone formation. To address this hypothesis, we performed *in vitro* studies using clonal wild-type and FAK−/− osteoblasts and assayed PGE_2_ release. Prostaglandins are released from bone cells as a result of mechanical stimulation [33] and are essential for load-induced bone formation *in vivo* [22]. We also transfected FAK−/− cells with wild-type FAK, Tyr-397, and Tyr-925 to determine importance of specific site mutations on osteoblast morphology, arrangement of cytoskeletal protein, and sensitivity to mechanical stimuli. Next, to determine whether these mechanisms are important *in vivo*, we subjected mice in which FAK was deleted in osteoblasts and osteocytes to three consecutive days of *in vivo* ulnar loading and analyzed bone formation rates and changes in bone geometry in response to axial compressive mechanical loading.

## Results

### FAK−/− osteoblasts exhibit reduced focal adhesion number relative to wild-type FAK osteoblasts

**Figure 1:**
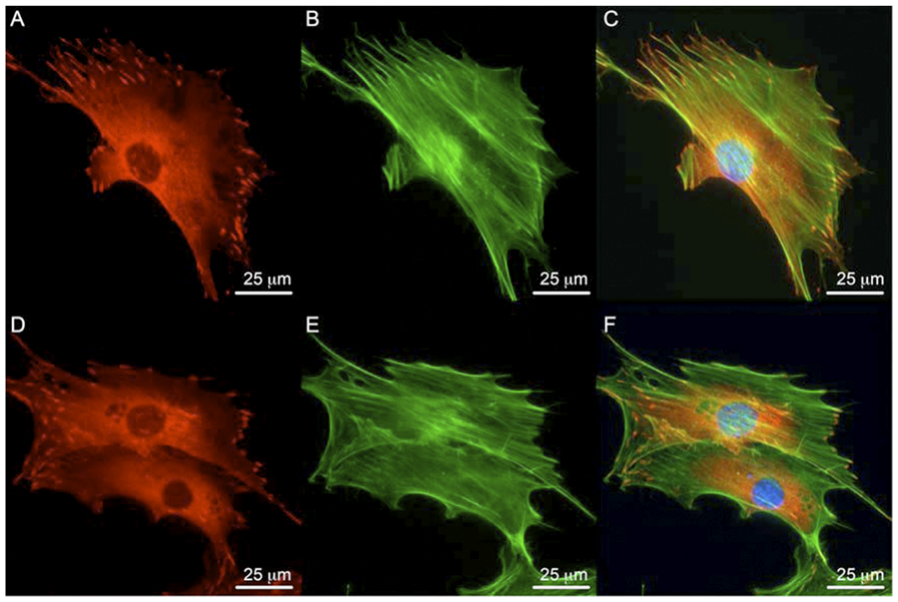
Focal adhesion formation and actin cytoskeleton in wild-type and FAK−/− osteoblasts. (A–C) FAK+/+ osteoblasts form focal adhesions (red) as shown by vinculin staining and display prominent actin fiber formation (green) as shown by phalloidin staining. (D–F) FAK−/− osteoblasts also exhibit actin fiber and focal adhesion formation. Panels C and F show merged images. DAPI nuclei stain in blue. Magnification = 60×. Scale bar represents 25 µm.

**Figure 2:**
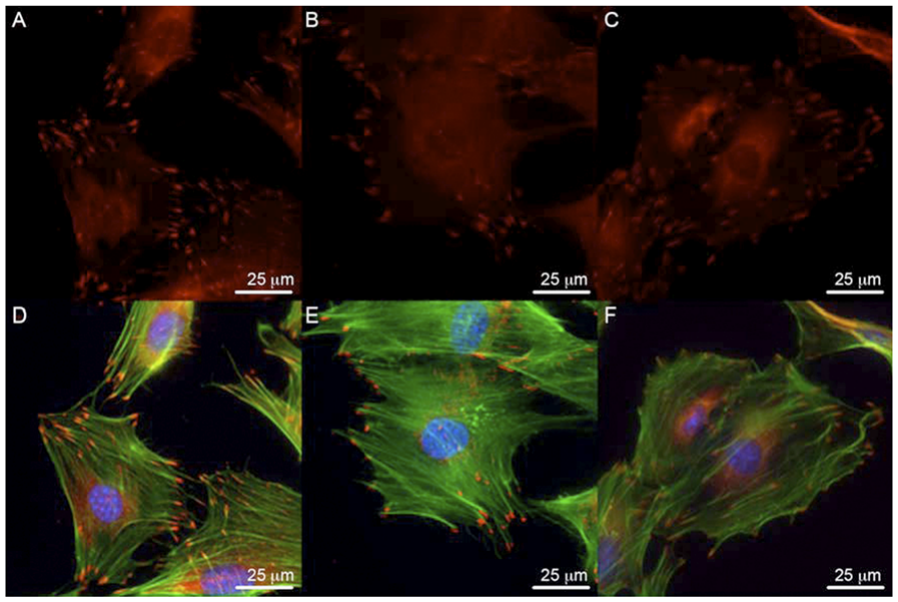
Paxillin expression and localization in osteoblasts. (A–C) Paxillin expression in MC3T3 osteoblasts, FAK+/+ osteoblasts and FAK−/− osteoblasts is similar. (D–F) Overlay of paxillin (red) and F-actin (green) as shown by phalloidin staining shows regions of strong paxillin expression and localization corresponding to the termini of actin fibers. FAK−/− cells exhibited fewer numbers of focal adhesions per cell area as quantified by point counting. DAPI nuclei stain in blue. Magnification = 60×. Scale bar represents 25 µm.

No differences in the actin cytoskeleton were observed (Figure 1). Vinculin expression and localization in FAK+/+ and FAK−/− osteoblasts were similar. Paxillin expression was observed in FAK+/+ and FAK−/− osteoblasts (Figure 2). The mean number of focal adhesions per cell area was significantly lower in FAK−/− osteoblasts (1.07×10^−3^+/−0.55×10^−3^; mean+/−SD) compared to FAK+/+ cells (2.84×10^−3^+/−1.27×10^−3^; mean+/−SD).

### Phosphorylated Pyk2 localizes to focal adhesions in FAK−/− osteoblasts

**Figure 3:**
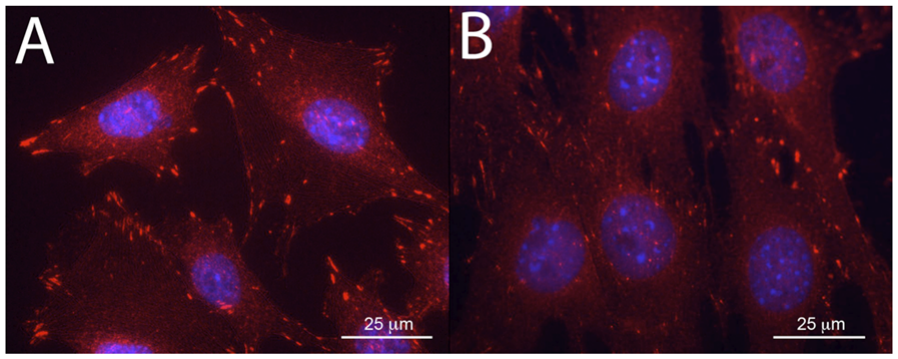
Expression of phosphorylated Pyk2 in FAK+/+ and FAK−/− osteoblasts. Expression and localization of phosphorylated Pyk2 at Tyr-402 (red) in FAK+/+ (A) and FAK−/− (B) osteoblasts. DAPI nuclei stain in blue. Magnification = 60×. Scale bar represents 25 µm.

Immunostaining for the activated form of Pyk2 (phosphorylated Tyr-402) is shown in Panels A and B of Figure 3. Panel A indicates localization of Pyk2 p-Tyr-402 to focal adhesions in FAK+/+ osteoblasts. FAK−/− osteoblasts also exhibited phosphorylated Pyk2 localized to focal adhesions, staining which appeared qualitatively more punctate but these observations were not quantified and further work is needed to explore changes in phosphorylated Pyk2 expression in the absence of FAK [24].

### Absence of FAK impairs flow-induced PGE_2_ release

**Figure 4:**
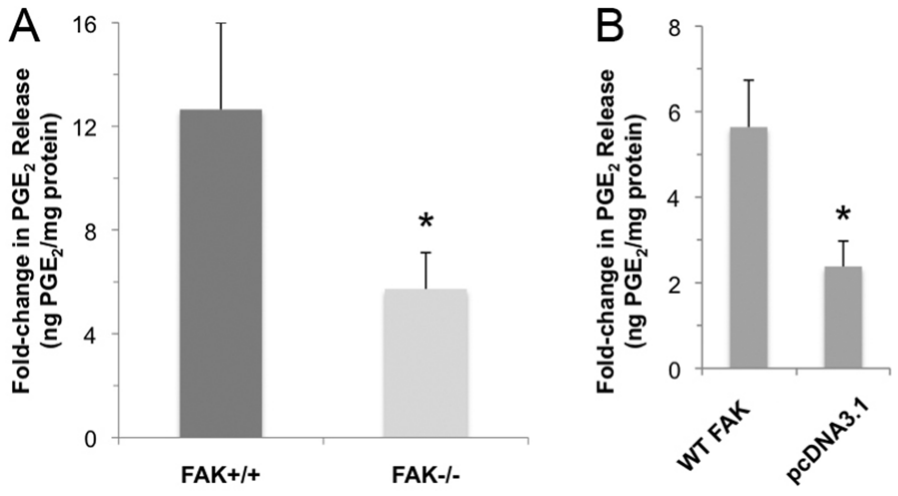
PGE_2_ release in response to oscillatory fluid flow. (A) Fold-change in PGE_2_ release in FAK+/+ and FAK−/− osteoblasts following 2 hours of oscillatory flow and a 4 hour post-flow incubation. FAK−/− osteoblasts exhibited a significantly lower fold-change in PGE_2_ release in response to fluid flow compared to FAK+/+ osteoblasts. (B) Fold-change in PGE2 release following fluid flow in mutant osteoblasts transiently re-expressing wild-type focal adhesion kinase and phosphospecific site mutations. Note that differences in absolute fold-changes in PGE2 release following flow in Panel A and Panel B represent variations in parallel assays.

Our results show that PGE_2_ release is activated immediately following flow with a maximum 12-fold increase in PGE_2_ release observed after 2 hours of flow and a 4 hour post-flow incubation (Figure 4, Panel A). FAK−/− osteoblasts exhibited only a ∼5-fold increase in response to flow, a difference that was significant (n = 9–10, *p*<0.05).

### Transient expression of FAK constructs partially restore flow-induced PGE_2_ release in FAK−/− osteoblasts

**Figure 5:**
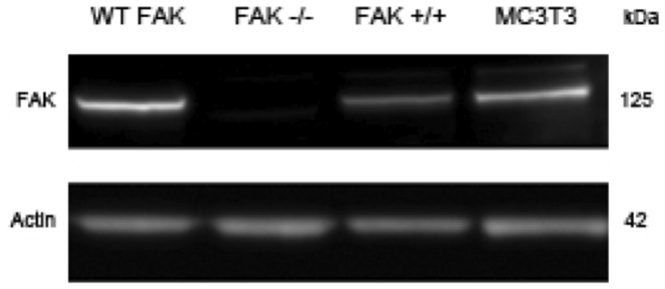
Western blot analysis of focal adhesion kinase expression in FAK−/− osteoblasts transiently transfected with the wild-type focal adhesion kinase (WT FAK) construct. Actin was used as a loading control. Expression in FAK−/−, FAK+/+, and MC3T3 osteoblast-like cells is shown as controls. WT FAK protein expression is similar to FAK expression in FAK+/+ and MC3T3 cells.

FAK−/− osteoblasts were transiently transfected with three FAK constructs (wild-type FAK, F397 and F925) and exposed to OFF. Restoration of wild-type focal adhesion kinase by transient transfection resulted in a significantly greater fold-increase in PGE_2_ release over the site-specific mutation constructs (n = 5–7, *p*<0.05) (Figure 4, Panel B). Cells transfected with site-specific mutations at Tyr-397 and Tyr-925 did not display any significant difference from the vector controls pcDNA3.1 (n = 5–7, *p*>0.05). Western blot analysis showed that FAK derived from the transiently transfected WT FAK construct was expressed at a level similar to FAK in FAK+/+ and MC3T3 cells (Figure 5).

### Body weight and geometric properties of long bones in FAK−/− mice

Characterization of 16-week-old animals revealed that the average body weight of WT and FAK−/− mice was within 1.2% for males and 8.7% for females (WT male = 42.9±5.8 g; FAK−/− male = 42.4±5.3 g; WT female = 34.6±5.6 g; FAK−/− female = 31.6±5.5 g; mean±SD), a difference that was not significantly different. Baseline characterization of geometric properties of the femur showed that I_MIN_, I_MAX_, cortical thickness and cortical area (Ct.Ar) at midshaft, and femur length, were not significantly different between genotypes (Table 1) in male and female mice. In addition, no genotype-based differences were detected in the trabecular microarchitecture of the distal femur in male and female mice (Table 2).

**Table 1:** Structural Properties of the Femur in WT and FAK−/− Adult Mice.

	Female		Male	
	*WT (n = 18)*	*FAK−/− (n = 32)*	*WT (n = 33)*	*FAK−/− (n = 15)*
Length, mm	16.11±0.47	16.04±0.61	16.47±0.40	16.45±0.46
I_MAX_ @ midshaft, mm^4^	0.287±0.061	0.314±0.013	0.434±0.087	0.420±0.086
I_MIN_ @ midshaft, mm^4^	0.166±0.037	0.164±0.045	0.239±0.060	0.219±0.038
Ct.Ar, mm^2^	1.11±0.13	1.19±0.16	1.37±0.14	1.35±0.15
Ct.Th, mm^2^	0.28±0.02	0.29±0.04	0.31±0.02	0.31±0.03

Mean ± SD; *n*, sample number; I_MAX_, maximum second moment of area; I_MIN_, minimum second moment of area; Ct.Ar, cortical area; Ct.Th, cortical thickness; ^a^ *p*<0.05 versus gender-matched WT group.

**Table 2:** Trabecular Microarchitecture in the Distal Femur of Adult WT and FAK−/− Mice.

	Female		Male	
	*WT (n = 18)*	*FAK−/− (n = 32)*	*WT (n = 33)*	*FAK−/− (n = 15)*
BV/TV	0.031±0.016	0.048±0.018	0.072±0.031	0.078±0.030
Tb. N, 1/mm	1.61±0.36	1.65±0.57	2.27±0.60	2.30±0.48
Tb.Th, mm	0.061±0.011	0.066±0.013	0.062±0.007	0.063±0.010
Tb.Sp, mm	0.661±0.153	0.674±0191	0.472±0.120	0.449±0.140
Conn.D	7.2±3.6	10.5±2.7	25.8±17.9	27.8±23.6
SMI	2.5±0.6	2.4±0.8	2.4±0.4	2.4±0.7

Mean ± SD; *n*, sample number; BV/TV, bone volume; Tb.N, trabecular number; Tb.Th, trabecular thickness; Tb.Sp, trabecular spacing; Conn.D, connectivity density; SMI, structure model index; ^a^ *p*<0.05 versus gender-matched WT group.

### Effect of conditional FAK deletion on mechanical adaptation of cortical bone

**Figure 6:**
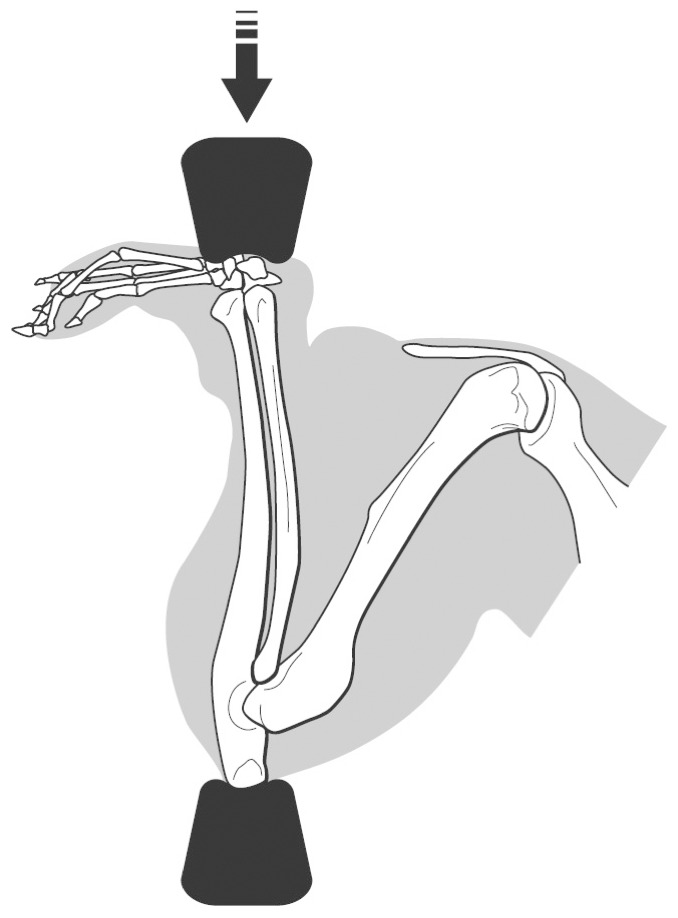
Rodent ulnar loading model. The right forearm in mice is subjected to cycle axial compression across the olecranon and flexed carpus while under isoflurane anesthesia. Due to the natural curvature of the ulna-radius complex in the medial direction, compressive loading creates a bending moment about the craniocaudal axis creating compressive and tensile bending strains on the medial and lateral surfaces, respectively.

**Figure 7:**
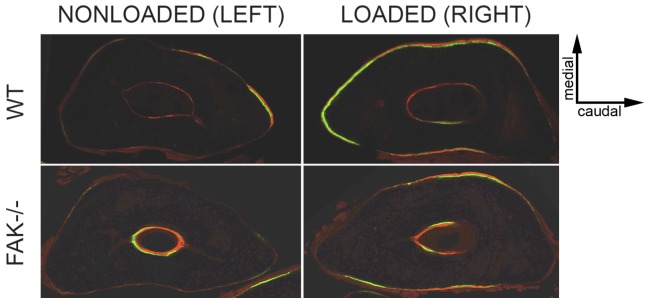
Transverse cross-sections at ulnar midshaft in nonloaded (left) and loaded (right) forearms in WT (top) and FAK−/− (bottom) mice given calcein (green) and alizarin (red) fluorochrome bone labels at 4 and 11 days, respectively, after the first day of loading. In response to applied mechanical loading, most new bone is formed on the medial and lateral surfaces where bending strains are highest. Note the appearance of double bone labels (green and red) on the medial and lateral surfaces of the ulna, as well as on the rostral surface, in the loaded ulna (top, right) compared to the nonloaded internal control (top, left) where very little bone formation is observed. The loaded ulna (bottom, right) in FAK−/− mice exhibit bone formation on the medial and lateral ulnar surfaces, but much less new bone formation, in terms of percent mineralizing surface, is observed relative to the nonloaded ulna (bottom, left). Sections are representative of the response observed for WT and FAK−/− mice. Magnification = 10×.

**Figure 8:**
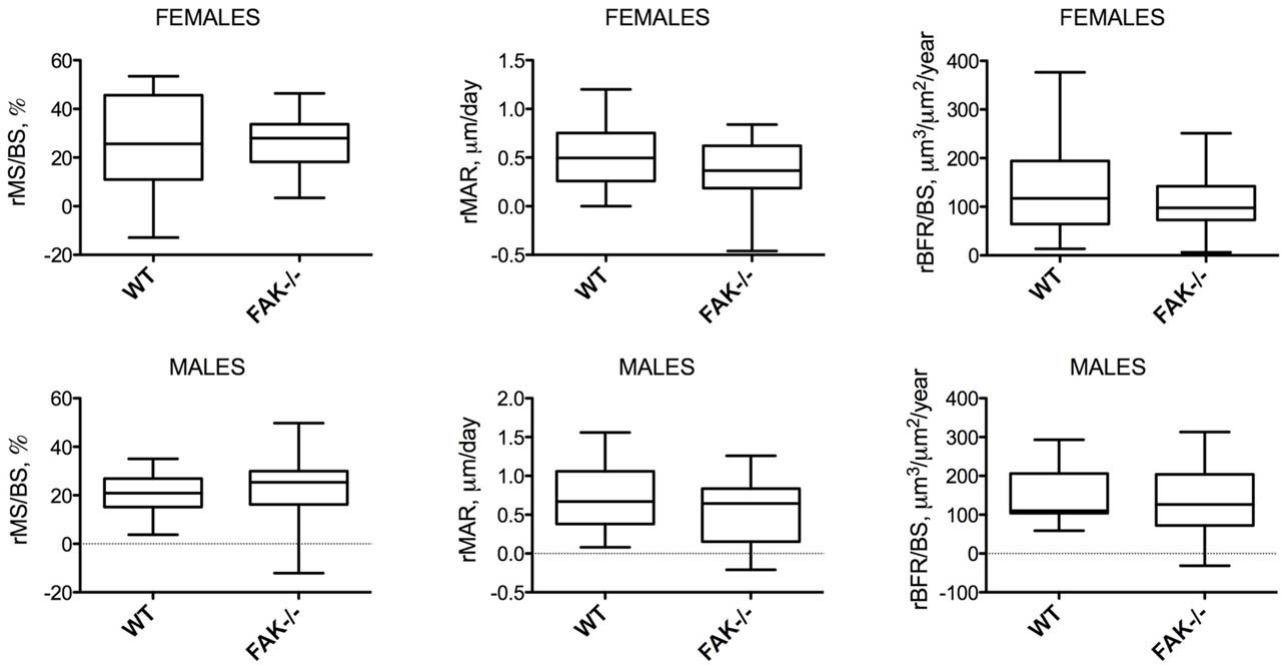
Relative bone formation parameters in WT and FAK−/− male and female mice. Conditional deletion of FAK did not affect relative mineralizing surface (rMS/BS), relative mineral apposition rate (rMAR) or relative bone formation rate (rBFR/BS), which is a product of rMS/BS and rMAR. Data are presented as box and whisker plots where the median, Q2, Q3 and whiskers, representing the 5% and 95% confidence intervals, are depicted.

To determine whether conditional deletion of FAK had an effect on mechanical adaptation of cortical bone *in vivo*, we subjected animals to three consecutive days of ulnar loading (Figure 6), and measured fluorescently-labeled newly-formed bone *in vivo*. We estimated strain levels during loading using a load-strain dose response curve determined in *ex vivo* bones, a procedure that has been previously described by Turner and colleagues [34]. Results from the load-strain calibration procedure showed that WT and FAK−/− mice experienced similar strains at the same load level. Males experienced peak mean strains of 3250±610 µε (WT) and 3468±495 µε (FAK−/−) at 3.0N and females experienced peak mean ulnar strains of 3190±610 µε(WT) and 3270±735 µε (FAK−/−) at 2.8N. Gender did not affect bone formation parameters (ΔImax, ΔImin, rMS/BS, rMAR, rBFR/BS), and combining male and female bone formation data did not change the outcome of the analysis. Here we report male and female data separately. FAK−/− mice, both male and female, exhibited significant increases in Imax and Imin in loaded ulna (Table 3). The percent increase, however, was not significantly different between gender-matched genotypes. In both WT and FAK−/− mice, loading resulted in new bone formation on the endosteal and periosteal bone surfaces (Figure 7). The maximum difference in the mean among bone formation parameters was 16% and was not statistically significant (Table 4, Figure 8) by a t-test (α = 0.05).

**Table 3:** Structural Properties of the Ulna in Response to High-Load.

	Female		Male	
	*WT (n = 16)*	*FAK−/− (n = 16)*	*WT (n = 11)*	*FAK−/− (n = 12)*
Length, mm	13.91±0.41	13.60±0.57	14.63±0.49	14.49±0.36
I_MAX_ (initial), mm^4^	0.0172±0.0063	0.0178±0.0048	0.0222±0.0046	0.0223±0.0063
I_MAX_ (final), mm^4^	0.0177±0.0063^a^	0.0180±0.0048^a^	0.0228±0.0046^a^	0.0228±0.0065^a^
ΔI_MAX_, %	2.64±2.08	1.50±0.90	2.83±2.22	2.23±1.93
I_MIN_ (initial), mm^4^	0.0036±0.0006	0.0041±0.0005	0.0043±0.0010	0.0044±0.0007
I_MIN_ (final), mm^4^	0.0041±0.0005^b^	0.0045±0.0005^b^	0.0049±0.0010^b^	0.0048±0.0008^b^
ΔI_MIN_, %	12.85±8.45	9.58±3.21	14.8±8.19	10.16±6.12

Mean ± SD; *n*, sample number; I_MAX_, maximum second moment of area; I_MIN_, minimum second moment of area; Δ, percentage change in I_MAX_ or I_MIN_ shown in gray rows;

a *p*<0.05 versus initial I_MAX_ value by a paired t-test;

b *p*<0.05 versus gender- and genotype-matched initial I_MIN_ value by a paired t-test.

**Table 4:** Ulnar Bone Formation Parameters.

	Female		Male	
	*WT (n = 16)*	*FAK−/− (n = 16)*	*WT (n = 11)*	*FAK−/− (n = 12)*
NL-MS/BS, %	14.3±12.3	12.1±9.8	14.6±9.8	18.7±17.8
L-MS/BS, %	39.6±17.4^a^	37.9±10.7^a^	35.9±7.7^a^	42.0±14.6^a^
rMS/BS, %	25.3±19.1	25.8±12.0	21.3±9.1	23.3±15.1
NL-MAR, µm/day	0.57±0.16	0.59±0.21	0.65±0.27	0.78±0.34
L-MAR, µm/day	1.11±0.34^b^	0.94±0.22^b^	1.40±0.38^b^	0.31±0.31^b^
rMAR, µm/day	0.54±0.35	0.35±0.32	0.74±0.44	0.53±0.42
NL-BFR/BS, µm^3^/µm^2^/yr	32.23±30.43	29.0±28.5	39.4±38.3	72.8±86.2
L-BFR/BS, µm^3^/µm^2^/yr	171.3±104.5^c^	133.7±53.2^c^	179.9±56.7^c^	207.0±113.4^c^
rBFR/BS, µm^3^/µm^2^/yr	139.0±99.6	104.7±56.4	140.5±70.5	134.2±97.6

Mean ± SD; *n*, sample number; NL, nonloaded; L, loaded; r, relative values (initial-final) are shown in gray rows; MS/BS, mineralizing surface; MAR, mineral apposition rate; BFR/BS, bone formation rate;

a *p*<0.05 versus gender- and genotype-matched NL-MS/BS by a paired t-test.

b *p*<0.05 versus gender- and genotype-matched NL-MAR by a paired t-test.

c *p*<0.05 versus gender- and genotype-matched NL-BFR/BS by a paired t-test.

## Discussion

FAK has been shown to be important in several cellular processes including migration [35], proliferation [36], and survival [37]. Global deletion of FAK is embryonic lethal [38], highlighting its important role in growth and development. Focal adhesions have been postulated to serve as mechanosensors in osteoblasts and osteocytes, and FAK is a key focal adhesion-associated signaling molecule implicated in mechanotransduction in bone cells. The main objective of this study was to determine the role of FAK in skeletal mechanotransduction. We hypothesized that FAK is critical in transducing mechanical forces into biochemical signals in the skeleton. We tested this hypothesis with FAK+/+ and FAK−/− osteoblast clonal lines in *in vitro* fluid flow studies to determine the precise role of FAK in osteoblast mechanotransduction. Deletion of FAK in osteoblasts *in vitro* was associated with fewer numbers of focal adhesions per cell area and reduced load-induced PGE_2_ release, an effect that was reversed with the introduction of a wild-type FAK construct, suggesting that FAK can mediate an osteogenic response in osteoblasts. The observation that transient expression of constructs harboring mutations at Tyr-397 and Tyr-925 do not lead to flow-induced PGE_2_ release suggests that these phosphorylation sites are involved in FAK-mediated mechanotransduction. One limitation of the present study is that mutant FAK protein expression levels following transient transfection of constructs could not be determined because anti-FAK antibodies did not recognize the mutant forms of the protein; however, transient transfection of the WT FAK construct into FAK−/− cells showed robust expression giving us a level of confidence that, in our hands, adequate levels of mutant protein expression are obtained.

We found that FAK cKO animals form bone with equal efficiency in response to mechanical loading compared to controls. While our *in vitro* data suggest that FAK is involved in osteoblast mechanotransduction, our *in vivo* loading data suggest that FAK is not required for load-induced bone formation in an intact system and presents the possibility of a signaling redundancy that compensates for a loss of FAK in osteoblasts and osteocytes.

Recent reports suggest an important role for FAK in reparative bone formation following skeletal injury. Using a mono-cortical tibial defect model, Kim et al. [24] showed that while progenitors were able to migrate to the site of skeletal injury and ultimately differentiate into mature osteoblasts, there was a delay in osteoblast differentiation and matrix formation in FAK−/− compared to WT animals. This difference was most evident 14 days post-surgery, but differences were lost by day 21. The authors concluded that integrin signaling via FAK was important for proper matrix deposition during healing, although deleting the FAK gene did not result in the complete abolition of matrix formation. Using a similar approach to study the role of FAK in load-enhanced reparative bone formation, Leucht et al. [25] applied a daily mechanical stimulus to a monocortical tibial implant during the healing process. Direct stimulation to bone marrow cells beneath the implant 1–3 days post-surgery and to the emerging bone regenerate 3+ days post-surgery enhanced osteogenic differentiation of bone marrow cells in WT animals during repair, but FAK−/− animals were unable to respond as robustly. They concluded that FAK inactivation blocked the ability of bone marrow cells to sense a mechanical stimulus and inhibited osteoblasts from forming matrix, suggesting an important role for FAK in mechanically enhanced bone repair.

One possible explanation for the discrepancy between our and their observations is that the ulnar loading model initiates new bone formation on the periosteal surface by activating progenitors residing in the periosteum, whereas, the tibial defect model triggers a bone healing process, which is comprised of several complex stages including inflammation, callus formation and remodeling. During the cortical repair process, a majority of the progenitor cells do not come from the periosteum but from the marrow or endosteum [39], [40] whereby progenitors must migrate to the site of injury before laying down new bone. Thus, the temporal and spatial requirements for FAK may be different in reparative versus load-induced bone formation and may involve other molecular mechanosensing mechanisms.

Recent *in vitro* studies suggest a role for FAK in bone cell mechanotransduction [18], [41], [42]. Young et al. [18] showed that disruption of FAK signaling in osteoblasts attenuated the load-induced activation of several key osteogenic outcomes including ERK phosphorylation, c-fos, Cox-2, PGE_2_ release, and osteopontin expression. Furthermore, the phenotype was rescued by re-expression of wild-type FAK. In a separate study, Santos et al. [42] reported that flow-induced activation of Wnt/β-catenin signaling in MLO-Y4 osteocytes was attenuated by the addition of FAK inhibitor-14. In addition, flow-enhanced proliferation of osteoblast-like cells was abolished with F397Y, a dominant-negative mutant of FAK [41] that blocks binding at the SH2 domain of Src-family non-receptor tyrosine kinases and Grb2 [43], both of which are important for integrin activated signaling.

We found that mechanically stimulated PGE_2_ release in FAK−/− osteoblasts was impaired in comparison to FAK+/+ osteoblasts, a result similar to that reported by Young et al. [18] supporting a role for FAK in osteoblast mechanotransduction. Cells expressing FAK harboring a mutation at two major tyrosine phosphorylation sites, Tyr-397 and Tyr-925, exhibited no such release, suggesting that these sites are essential in mediating PGE_2_ release following fluid flow. That is, autophosphorylation at Tyr-397 within the N-terminal domain and phosphorylation of Tyr-925 within the focal adhesion targeting C-terminal domain are important in transmitting external mechanical stimuli into osteogenic signals.

The presence of an intact cytoskeleton in the absence of focal adhesion kinase, even though the number of focal adhesions is reduced, still permits transmission of forces sensed at the points of cell-surface contact and permits other signaling molecules to compensate for the loss of FAK. This may explain the unaltered osteogenic response to *in vivo* loading observed in FAK−/− mice. Indeed, recent data suggest that Pyk2 may compensate for loss of FAK in various cell types including endothelial cells [44] and fibroblasts [45]. Others [24] have shown that phosphorylated Pyk2 was expressed more robustly in FAK−/− osteoblasts at focal adhesions by immunofluorescence, suggesting enhanced Pyk2-focal adhesion interactions. Others have reported that Pyk2 expression levels are not altered in FAK−/− osteoblasts [18]; however, the change in localization of Pyk2 to focal adhesions may be sufficient to restore some functional activity to integrin-mediated mechanotransduction. In fact, Pyk2−/− mice exhibit increased bone formation due to disruption of osteoclast function [46] and repression of osteoblastic activity [47]. Furthermore, Turner and colleagues have shown that Pyk2 knockout mice exhibit greater load-induced bone formation rates compared to wild-type controls [48], suggesting that Pyk2 may normally repress an adaptive response to mechanical stimuli. While we did not definitively show that phosphorylated Pyk2 expression levels are altered in FAK−/− osteoblasts, expression qualitatively appeared more punctate. Additional data are needed to determine the precise role of Pyk2 in bone mechanotransduction *in vivo*.

In summary, we showed that FAK−/− osteoblasts were less responsive to mechanical stimulation *in vitro* as measured by PGE_2_ release, and that Tyr-397 and Tyr-925 phosphorylation sites conferred functional activity; however, deletion of FAK in osteocytes and osteoblasts *in vivo* had no significant effect on the ability of mice to form bone in response to mechanical loading. These results challenge the current hypothesized role for FAK in bone mechanotransduction *in vivo* and suggest that alternative signaling molecules mediate integrin-specific signal transduction in mechanical adaptation of the skeleton.

## Materials and Methods

### Ethics statement

All work was performed in accordance with the guidelines of the American Association for the Accreditation of Laboratory Animal Care. In addition, the Palo Alto Veterans Affairs Medical Center Institutional Animal Care and Use Committee approved all procedures (IACUC No. CAT1350 CAT090501MOU, approved 03/27/12).

### Bone cell culture

Primary immortalized osteoblastic clonal lines expressing native wild-type FAK (FAK^+/+^ clone 1E11) and a FAK−/− mutation (FAK^−/−^ clone ID8) were previously established as described in Kim et al. [24]. All cells were maintained at 37°C with 5% CO_2_ in complete growth media (MEMα+ 10% FBS +1% Penicillin-Streptomycin). Cells were passaged using 0.05% trypsin (Invitrogen). Passages 7 to 9 were used in all experiments.

### Transient DNA transfections

FAK−/− osteoblasts were transiently transfected with three FAK plasmids separately using Lipofectamine 2000 reagent (Invitrogen). An empty vector was used as a negative control. The FAK plasmids consisted of wild-type FAK [pcDNA3.1/Hygro(+)-WT], FAK with a site specific mutation at Tyr-397 [pcDNA3.1/Hygro(+)-F397 FAK], and FAK with a site specific mutation at phosphorylation site Tyr-925 [pcDNA3.1/Hygro(+)-F925 FAK]. FAK−/− osteoblasts were plated at 70–75% confluence onto 100 mm tissue culture dishes and grown overnight in antibiotic free media prior to transfection. The conditions for transfection were optimized by varying the ratio of plasmid DNA (µg) to the volume of Lipofectamine 2000 (µl) from 1∶1 to 1∶4 in Opti-MEM media (Invitrogen). The optimal transfection ratio (20 µg of plasmid DNA per 40 µl of Lipofectamine 2000 reagent) was determined by Western blot analysis for FAK protein expression. At 24 hours post-transfection, cells were used for described experiments.

### Antibodies

Primary antibodies used were anti-mouse monoclonal FAK antibody (clone 4.47) (Millipore), rabbit polyclonal antibody against phosphospecific FAK Tyr-397 (Santa Cruz Biotechnology), rabbit polyclonal antibody against phosphospecific p-proline-rich tyrosine kinase 2 (Pyk2)-Tyr 402 conjugated to Alexa Fluor 488 (Invitrogen), chicken antibody against paxillin (clone 349) (BD Biosciences), monoclonal anti-vinculin (clone hVIN-1) (Sigma) and actin mouse monoclonal antibody (clone AC-40) (Abcam). Secondary antibodies used were goat anti-rabbit-HRP (Santa Cruz Biotechnology), goat anti-mouse-HRP (Santa Cruz Biotechnology), anti-mouse monoclonal FAK (clone 4.47) conjugated to Alexa Fluor 555 (Upstate), phalloidin-conjugated Alexa Fluor 488 (Invitrogen) and goat anti-mouse Alexa Fluor 568 (Invitrogen).

### Western blot analysis

Expression of transiently transfected WT FAK construct was verified by Western blot. Cells were rinsed twice in cold PBS and lysed using RIPA lysis buffer (Santa Cruz Biotechnology). Cell lysates were sheared using a 21 gauge needle and centrifuged for 10 min at 16,000 rpm. The protein concentration of the resultant supernatants was quantified by bicinchoninic acid analysis (BCA) (Pierce Chemical). Protein was denatured for 10 min at 70°C, and 10–20 µg per lane were loaded into a NuPAGE 4–12% Bis-Tris polyacrylamide gel (Invitrogen) for separation and transferred to nitrocellulose for immunoblotting. Nonspecific binding sites were blocked with 5% bovine serum albumin (Sigma) in 1% Tris-buffered saline solution containing 0.1% Tween 20 (TTBS) for 2 hours at room temperature. After blocking, immunoblots were probed with FAK and actin antibodies diluted in 5% BSA/TTBS overnight at 4°C at a 1∶1000 dilution and then incubated with goat anti-mouse (1∶4000) and goat anti-rabbit (1∶8000) antibodies conjugated to horseradish peroxidase (HRP) for one hour at room temperature. HRP was detected using Immun-Star WesternC Chemiluminescence (Bio-Rad) and quantified using an automated imager (LAS-4000, Fujifilm).

### Immunofluorescence staining

To examine cellular morphology and localization of FAK, vinculin, talin, paxillin and Pyk2, MLO-Y4 osteocytes, MC3T3 osteoblasts, and wild-type FAK and FAK−/− osteoblasts were seeded onto fibronectin-coated cover slips and cultured overnight in 24-well plates. The cover slips were rinsed twice in PBS, fixed in 4% paraformaldehyde, and permeabilized in 0.1% Triton-X (Sigma). The samples were incubated in primary blocking solution containing 1% BSA in PBS (1% BSA/PBS) for 1 hour at room temperature. The cell-seeded cover slips were probed for focal adhesion kinase, vinculin, talin, or paxillin followed by the appropriate secondary antibodies (see *Antibodies*). Cells were imaged on an Olympus IX71 microscope mounted with a Hamamatsu C9100 digital camera. Images were captured and processed using MetaMorph software (Molecular Devices). The number of focal adhesions was quantified in wild-type FAK and FAK−/− osteoblasts. Focal adhesions were visualized by paxillin localization in individual cells and quantified by manual point-counting using Image J software. Individual cell area was determined by phalloidin-stained actin using a thresholding technique.

### Oscillatory fluid flow (OFF)

An *in vitro* model of fluid flow in bone [49] was used to investigate the role of focal adhesion kinase in bone cell mechanotransduction. FAK^+/+^ and FAK−/− osteoblasts were cultured on fibronectin-coated (10 µg/ml) glass slides at a density of approximately 2–3×10^5^ cells/slide. Cells were incubated for 24 to 48 hours and serum starved in reduced serum media (MEMα+ 0.5% FBS +1% Penicillin-Streptomycin) overnight. At 80% confluence, cells were loaded into parallel-plate flow chambers as previously described [49]. Cells were exposed to laminar oscillatory fluid flow at a peak shear stress of 1 Pa (10 dynes/cm^2^) and a frequency of 1 Hz for 5, 30 and 120 min to determine the time course of mechanically-induced PGE_2_ release. The chambers were placed in the incubator at 37°C with 5% CO_2_ throughout the duration of flow to maintain a constant temperature and pH. Static control samples were maintained in 100 mm tissue culture dishes at the same conditions. After flow, slides were immediately removed from the flow chambers and prepared for PGE_2_ release.

### Prostaglandin E_2_ (PGE_2_) release

Peak levels of PGE_2_ release from FAK+/+ and FAK−/− osteoblasts were determined using ELISA. Immediately following OFF, slides were removed from the flow chambers, rinsed with warm PBS, placed in sterile tissue culture dishes, and covered with approximately 1 ml of fresh flow media. Following an incubation period of 4 or 24 hours at 37°C and 5% CO_2_, media was collected and assayed. A PGE_2_ Enzyme Immunoassay (EIA) Kit (Assay Designs) was used to measure PGE_2_ concentrations in media samples per the manufacturers instructions.

### Animals

The Palo Alto Veterans Affairs Medical Center Institutional Animal Care and Use Committee (IACUC) approved all experimental procedures. Global deletion of FAK results in embryonic lethality [50], therefore we deleted FAK specifically in osteoblasts and osteocytes by crossing mice with conditional floxed FAK alleles [24], [51] (gift from Jill Helms, Stanford University) and transgenic mice harboring the Cre gene under the control of the 2.3-kb proximal fragment of the α1(I) collagen promoter [52] to generate experimental animals [FAK^flox/flox^;Col2.3 α 1(I)-Cre^−^ (WT) and FAK^flox/flox^;Col2.3 α 1(I)-Cre^+^ (FAK−/−)] in which FAK was ablated in osteoblasts and osteocytes [51]. Offspring were on a mixed FVB/C67BL/6 background. Cre expression under the control of the 2.3-kb proximal fragment of the α1(I) collagen promoter is observed at high levels in osteoblasts and osteocytes [52] and results in effective deletion of FAK with a Cre-LoxP approach [24]. Previous studies show that Col2.3-Cre animals express Cre specifically in osteoblasts and osteocytes [52], and osteoblast/osteocyte deletion of FAK in the FAKfloxed::Col2.3-Cre offspring has been confirmed previously by *in situ* hybridization [24].

Offspring were genotyped using DNA from tail clips and primers P1(F): 5′-GAC CTT CAA CTT CTC ATT TCT CC-3′, P2(R): 5′-GAA TGC TAC AGG AAC CAA ATA AC-3′ and P3(F): 5′-GAG AAT CCA GCT TTG GCT GTT G-3′ [51] yielding a 327 bp fragment for the Cre-mediated recombined region (P1/P2), a 290 bp fragment for the WT allele (P2/P3) and a 400 bp fragment for the floxed FAK gene (P2/P3). FAK^flox/flox^;Col2.3α1(I)-Cre- and FAK^flox/flox^;Col2.3α1(I)-Cre+ male and female animals were used in loading experiments. Siblings were housed in groups of up to five animals at the VAPA Medical Center Veterinary Unit where they had *ad libitum* access to standard mouse chow and water. Animals were weighed at 16 weeks of age and body weight was recorded.

### Micro-computed tomography (µCT)

Trabecular bone microarchitecture in the distal femur and cortical bone geometry at the femur midshaft were evaluated using high-resolution computed tomography (vivaCT40, Scanco Medical). The femur was chosen for analysis because it is a load-bearing bone and its distal region provides enough trabecular tissue for analysis of basal levels of trabecular bone in the adult skeleton. Bones were secured in a custom sample holder and scanned transverse to the long axis using a 55 kVp energy x-ray source and 2048×2048 pixel slices with a 10.5 µm isotropic voxel size. The slices were reconstructed using the manufacturer's software. From the resulting grayscale images, a region of interest (ROI) was manually outlined in each CT slice encompassing trabecular bone. For the distal femur, the ROI started at approximately 0.25 mm proximal to the distal femoral growth plate and spanned ∼4 mm (400 slices) proximally. The edge of the ROI in individual slices was manually drawn at approximately 0.1 mm from the endocortical surface. The slices were evaluated at a fixed threshold to distinguish bone tissue from surrounding soft tissue. Approximately 200 transverse slices were evaluated for morphometric parameters including bone volume fraction (BV/TV, %), trabecular number (Tb.N, 1/mm), trabecular thickness (Tb.Th, mm), trabecular spacing (Tb.Sp, mm), connectivity density (Conn.D), and structure model index (SMI). SMI is a measure of the structure of cancellous bone and relates to the trabecular convexity. An ideal (flat) plate, cylinder and sphere have SMI values of 0, 3 and 4, respectively [53]. An ROI spanning ∼1 mm was scanned at the femur midshaft, and approximately 50 slices were used to calculate cortical area (Ct.Ar), cortical thickness (Ct.Th), and moments of inertia I_MIN_ and I_MAX_ at the femur midshaft using Scanco software.

### *In situ* ulnar strain analysis

Mechanical strains achieved during loading were measured as described previously [54] using 16-wk-old animals from each experimental group (n = 5 per group). Briefly, immediately following euthanasia (isoflurane to effect and cervical dislocation) the forelimbs were dissected free at the shoulder, keeping the soft tissue surrounding the forearm intact. The musculature was retracted exposing only the medial diaphysis of the ulna, and a 120 Ω single-element strain gauge (EA-06-015DJ-120, Vishay Measurements Group) was bonded to the surface with cyanoacrylate adhesive centered approximately 3.5 mm distal to the insertion point of the brachialis muscle. Previous studies showed that this was the most reliable method of positioning the gauge at the ulnar midshaft [34]. Each gauge was conditioned with a 0.8 V bridge excitation voltage and amplified with a gain of 300× using a signal conditioner (Model 2210, Vishay Measurements Group). The amplified analog strain signals were digitized using an AD-DA board (aISA-A57, Adtek-System Science, Kanagawa, Japan). For the strain calibration procedure, each forearm was axially loaded (Figure 6) using a Bose ElectroForce® 3200 mechanical testing machine at increasing peak load levels (1N, 1.5N, 2.0N, 2.5N and 3.0N). Voltage data for each of the loading waveforms were converted to strain values using a conversion factor determined by electronic shunt calibration of the measuring hardware and confirmed by calculated strains using an aluminum cantilever.

### *In vivo* ulnar loading

Sixteen-week-old experimental male and female mice were divided into low, medium and high loading groups. Each group was subjected to axial cyclic ulnar loading while under isoflurane anesthesia (Baxter International) using the mechanical testing system described above. Due to the natural curvature of the ulna in the medial direction, loading in this manner causes bending about the craniocaudal axis creating compressive and tensile bending strains on the medial and lateral surfaces, respectively [34]. Thus, most new bone formation occurs on the medial and lateral surfaces where bending strains are highest. The applied force was controlled via load feedback using a 50 g load cell (Honeywell Sensotec). The right forearm was loaded on three consecutive days, while the left ulna served as an internal nonloaded control. Peak loads were 3.0N for males and 2.8N for females, which correspond to approximately 3500 µε. All animals were given *in vivo* sequential bone labels at 7 (calcein, 30 mg/kg, IP) and 14 (alizarin, 50 mg/kg, IP) days after the first day of loading. Fluorochrome labels bind to calcium ions and are incorporated into newly forming matrix on bone surfaces. When labels are administered at different times, the amount of new bone formed between label administration times can be quantified using standard dynamic histomorphometric techniques.

At sacrifice, the right and left ulnae were harvested, fixed in 10% neutral buffered formalin for 48 h and stored in 70% ETOH at 4°C until further processing.

### Histomorphometry

Right and left ulnae were dehydrated in sequential ascending concentrations of ethanol (70, 80, 90 and 100%) and embedded undecalcified in methylmethacrylate (Sigma) as described previously [55]. Three 90-µm thick sequential transverse sections were cut at the midshaft using an Isomet Precision Saw (Buehler Ltd.). The sections were ground to a final thickness of 50 µm and then mounted unstained on standard microscope slides. One section per ulna was analyzed at a magnification of 10× using a Nikon TE-2000/C1 confocal microscope (Nikon, Inc.). ‘Measured’ histomorphometric variables on the periosteal surface were obtained using ImageJ, and dynamic bone formation indices calculated. Variables measured included total bone perimeter (B.Pm, mm), single label perimeter (sL.Pm, mm), double label perimeter measured along the innermost label (dL.Pm, mm), and the interlabel width (Ir.L.Wi, µm), which is the distance between the first and second labels [56]. When only single labels were present on a bone section, mineral apposition rate was estimated as the minimum value observed in that particular experimental group [55]. ‘Derived’ or calculated variables were mineralizing surface (MS/BS = 100*[0.5*sL. Pm+dL.Pm]/B.Pm, %), mineral apposition rate (MAR = dL.Ar/dL.Pm/days between labels, µm/day) and bone formation rate (BFR/BS = MAR*[MS/BS]*3.65, µm^3^/µm^2^/year). Relative values (rMS/BS, rMAR, and rBFR/BS) for each animal were calculated by subtracting nonloaded (left) from loaded (right) values to control for individual differences between animals.

### Structural properties of the ulna

Load-induced changes in cortical cross sectional geometry are represented by changes in maximum and minimum second moments of area (I_MAX_, I_MIN_), measures of resistance to bending about two perpendicular principal axes in the plane of the bone section. One section from the loaded (right) ulnar midshaft was imaged at a magnification of 10×. Initial and final cortical bone geometry was determined by observing bone labels administered at the beginning and end of the loading period as previously described [55]. Images of initial and final cross-sections were imported into ImageJ (Scion Corporation), and I_MIN_ (mm^4^) and I_MAX_ (mm^4^) were calculated for each image using a customized macro. Percent change in I_MIN_ and I_MAX_ before and after loading [100*(I_final_−I_initial_)/I_initial_] was calculated [55]. I_MIN_ and I_MAX_ were calculated directly from sections because it is only by visualizing bone labels that one can distinguish between cortical geometry both pre- and post-loading.

### Data analysis

*In vivo* data were checked for normality and constancy of variance. The effects of gender and genotype on all outcome measures were analyzed by a two-factor ANOVA. When a significant effect of gender was detected, male and female data were analyzed separately. Estimated peak ulnar strain was calculated for each genotype using a load-strain calibration procedure. Unpaired t-tests (SPSS® Base 16.0 Statistical Software) were used to assess differences in males and females separately based on genotype in body weight, structural properties of the femur, and relative (loaded minus nonloaded) bone formation parameters in the ulna. Effects of mechanical loading were determined by comparing histomorphometric parameters and structural properties from loaded (right) and nonloaded (left) ulnae using a paired t-test. Statistical significance was assumed for *p*<0.05. Our experimental design yielded a minimum detectable difference of mean of 0.77 standard deviations with a statistical power of 80%. Data are presented as mean ± standard deviation (SD) except for relative bone formation parameters, which are presented as box-and-whisker plots showing the median and 95% confidence intervals. *In vitro* quantitative data are presented as the mean ± standard error (SE). A one-way ANOVA followed by a Bonferroni post hoc analysis (SPSS) was used to test for differences in PGE2 release between multiple groups. For two-sample comparisons, an unpaired Student's t-test was used. Statistical significance was assumed for *p*<0.05.
